# A study on respiratory management in acute postoperative period by nasal high flow for patients undergoing surgery under general anesthesia

**DOI:** 10.1097/MD.0000000000021537

**Published:** 2020-07-31

**Authors:** Shinji Kurata, Gaku Mishima, Motohiro Sekino, Shuntaro Sato, Maximilian Pinkham, Stanislav Tatkov, Takao Ayuse

**Affiliations:** a Department of Dental Anesthesiology; b Division of Intensive Care; c Clinical Research Center, Nagasaki University Hospital, Nagasaki, Japan; d Fisher and Paykel Healthcare Ltd, Auckland, New Zealand; e Division of Clinical Physiology, Department of Translational Medical Sciences, Nagasaki University Graduate School of Biomedical Sciences, Nagasaki, Japan.

**Keywords:** nasal high flow, postoperative acute period, postoperative hypercapnia, postoperative hypoxemia

## Abstract

In head and neck surgery where the oropharyngeal area is the operative field, postoperative respiratory depression and upper airway obstruction are common. Therefore, supplemental oxygen is administered to prevent severe postoperative early hypoxemia. However, a high concentration of oxygen increases the likelihood of secondary complications, such as carbon dioxide (CO_2_) narcosis. Nasal high-flow (NHF) therapy generates high flows (≤60 L/min) of heated and humidified gas delivered via nasal cannula and provides respiratory support by generating positive airway pressure, clearance of dead space and reduction of work of breathing. This study aims to determine whether the postoperative hypoxemia and hypercapnia can be prevented by NHF without the requirement of supplemental oxygen. The study will recruit adult patients undergoing planned oral surgery under general anesthesia at Nagasaki University Hospital. It is a randomized parallel group comparative study with 3 groups: NHF with room air only and no supplemental oxygen, no respiratory support, and face mask oxygen administration. The study protocol will begin at the time that the patient is returned to the general ward and will finish 3 hours later. The primary endpoint is the time-weighted average of transcutaneous O_2_ over the 180 minutes and secondary endpoints are the time-weighted average of transcutaneous CO_2_ (tcpCO_2_), SpO_2_, and respiratory rate, incidence rate of marked hypercapnia (tcpCO_2_ ≥60 mm Hg for 5 minutes or longer), incidence rate of moderate hypercapnia (tcpCO_2_ ≥50 mm Hg for 5 minutes or longer) and the percentage of time that SpO_2_ is <90%. Included also is a group in which the postoperative management is performed only by spontaneous breathing without performing respiratory support such as oxygen administration, to investigate the efficacy and necessity of conventional oxygen administration. This exploratory study will investigate the use of NHF without supplemental oxygen as an effective respiratory support during the acute postoperative period.

**Trial registration:**

The study was registered the jRCTs072200018. URL https://jrct.niph.go.jp/latest-detail/jRCTs072200018

## 1 Introduction

Respiratory failure is the most common postoperative pulmonary complication. In head and neck surgery where the oropharyngeal area is the operative field, postoperative respiratory depression and upper airway obstruction are particularly common. Routine oxygen administration aims to prevent this postoperative early hypoxemia. Furthermore, it may be recommended to administer oxygen with a certain allowance, such as until the next morning if the operation is highly invasive or if the operation end-time is during the night-shift with reduced staff numbers. Therefore, there is increased likelihood of administering greater levels of oxygen in these patients than required. The routine administration of oxygen does not address the problem of hypoventilation, may lead to CO_2_ retention and may delay the discovery of worsening respiratory function.^[1]^

Nasal high flow (NHF) is drawing attention as a respiratory support therapy. NHF generates high flows (up to 60 L/min) of heated and humidified gas delivered via nasal cannula. NHF provides respiratory support by generating positive airway pressure, clearance of dead space, reduction of work of breathing and improved mucociliary clearance.^[2–5]^ NHF can be delivered as room air only or with supplemental oxygen, depending on the context.

In a recent study, NHF with room air and no supplemental oxygen was effective as a respiratory support method under spontaneous breathing during moderate propofol sedation.^[6]^ It has been reported that transcutaneous CO_2_ partial pressure and SpO_2_ can be maintained in the normal range by reducing respiratory effort. Therefore, it is possible that NHF without the combined use of oxygen can be an effective therapy for patients during the postoperative acute phase. If NHF with room air and no supplemental oxygen can prevent postoperative early hypoxemia, the authors believe that safer respiratory management will be possible in general wards.

This research aims to determine whether postoperative hypoxemia and hypercapnia can be prevented by NHF. The authors’ hypothesis is that an application of NHF without oxygen supplement may prevent hypoxemia and hypercapnia after surgery under general anesthesia. Furthermore, the study is designed to investigate whether spontaneous breathing alone with no respiratory support is safe, therefore testing the efficacy and necessity of conventional oxygen administration. As there have been no previous studies related to this purpose, there is no basis for calculating the sample size. Therefore, this research will be conducted as an exploratory exercise, and the main of full-scale clinical study will be carried out as the next stage based on the results. Through this study and the verification studies planned after that, if hypoxemia and hypercapnia can be prevented by using NHF in the postoperative acute phase, safer and more comfortable acute respiratory management will be possible.

## 2 Methods/design

### 2.1 Study design

The present study was designed in accordance with the Standard Protocol Items: Recommendations for Interventional Trials and Consolidated Standard of Reporting Trials 2010 guidelines.^[7,8]^ This will be a randomized controlled trial (RCT) in patients scheduled for oral-maxillofacial surgery under general anesthesia.

The Clinical Research Review Board at Nagasaki University approved the study. The trial will be conducted at Nagasaki University Hospital in Japan and is registered on the RCTs. The study will be conducted in accordance with the principles of the Declaration of Helsinki and the established best clinical practices of Japan.

This is an exploratory study and therefore, the sample size is set based on the feasibility in the hospital. For efficacy purposes, the time-weighted average value of transcutaneous CO_2_ partial pressure (tcpCO_2_), transcutaneous O_2_ partial pressure (tcpO_2_), SpO_2_, and respiratory rate during the observation period will be evaluated.

### 2.2 Participant recruitment

**Figure 1:**
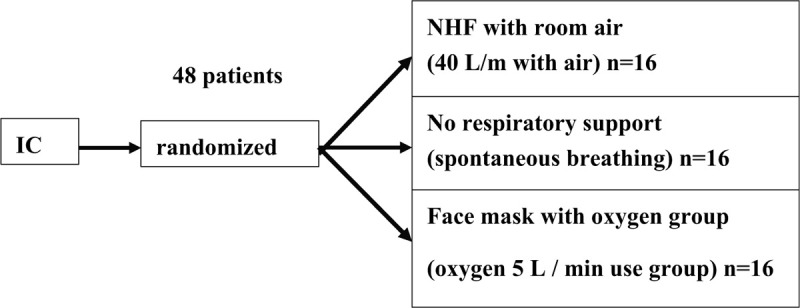
After obtaining informed consent, 48 subjects who meet the registration requirements will be randomly assigned to either of the 3 groups. The experimental group will be: Experimental group - NHF with room air only and no supplemental oxygen; Control group 1 - no respiratory support; Control group 2 - face mask oxygen administration (oxygen 5 L/min use). NHF = nasal high flow.

The study will recruit adult patients undergoing planned oral surgery under general anesthesia at Nagasaki University Hospital. It is a randomized parallel group comparative study with 3 groups. After obtaining informed consent, the target patients who meet the registration requirements will be randomly assigned to one of the 3 groups (ratio 1:1:1). The groups are as follows: Experimental group - NHF with room air only and no supplemental oxygen; Control group 1 - no respiratory support; Control group 2 - face mask oxygen administration (oxygen 5 L/min use) (Fig. 1).

### 2.3 Inclusion criteria

Target patients who meet all of the following criteria:

1. Patients scheduled for oral-maxillofacial surgery under general anesthesia
2. Patients who have been given sufficient information about the study and have provided their full understanding and consent to the patient's free will
3. At the time of obtaining consent, an adult patient between 20 and 85 years old

### 2.4 Exclusion criteria

Patients who meet at least one of the following are to be excluded:

1. Patients who have already been receiving continuous oxygen (home oxygen therapy)
2. Patients who cannot breathe through their nose
3. Patients who cannot reduce or discontinue antithrombotic drugs after surgery
4. Patients with a history of pneumothorax
5. Patients who the investigator judged to be inappropriate for the study
6. Severe acute respiratory syndrome coronavirus polymerase chain reaction test positive patients

#### 2.4.1 Sample size

The study is exploratory and aims to collect information for conducting verification research. Therefore, the sample size is set based on the feasibility. There are about 240 patients who undergo oral surgery within 5 hours under general anesthesia at Nagasaki University Hospital in 1 year. If we estimate that about 60% of the cases in the 4-month (80 cases) are available for obtaining consent, target sample size is 48. The 48 cases of oral surgery will be divided into the 3 groups: NHF with room air, 16 cases; face mask oxygen administration, 16 cases; and no respiratory support, 16 cases.

### 2.5 Study protocol

The study protocol will begin at the time that the patient is returned to the general ward and will finish 3 hours later. Patients assigned to the NHF group will be placed on NHF therapy with a starting flow rate of 40 L/min at the time of returning to the general ward. As with normal respiratory management in the general ward, if SpO_2_ shows a value of 90% or less due to a factor other than body movement during NHF use and the monitor alarm is activated, then supplemental oxygen may be applied until SpO_2_ returns to above 90%. Patients may request to remove the NHF cannulas, at which time they may switch to oxygen administration by face mask.

Patients assigned to the face mask oxygen administration group will be started on 5 L/min supplemental oxygen (FiO_2_ around 0.4) via face mask starting at the return to the general ward until 3 hours later. The level of supplemental oxygen will be adjusted to maintain SpO_2_ above 90%.

In the group that will not receive respiratory support upon arrival in the ward, each patient will be monitored and if the SpO_2_ remains at ≤90% then the patient will be placed on face mask oxygen administration, and administration of oxygen will start at 5 L/min until SpO_2_ returns to the normal value above 90%.

After the operation, the vital signs of the patient will be evaluated, including blood pressure, electrocardiogram, and SpO_2_, which are the usual measurements in general wards. Separate to these assessments, the partial pressure of tcpCO_2_, partial pressure of tcpO_2_ and respiratory rate will be continuously monitored. To continuously measure the breathing parameters, respiratory inductance plethysmography will be used (QDC-Pro, made by CareFusion). The chest and abdominal movements is traced, and analog data will be output externally and recorded in PowerLab. In addition, the tidal volume, respiratory rate, and minute ventilation are calculated from the measured data.

### 2.6 Adverse events

During the study, it is possible that the NHF interface or oxygen face mask may cause discomfort. Moreover, an electrode for transcutaneous measurement of oxygen and CO_2_ concentration will be attached to the anterior chest. For participants with sensitive skin, this might cause temporary redness. If and when these adverse events occur, care will be taken to prevent undue harm to the research subjects.

### 2.7 Outcome

The primary endpoint is: time-weighted average of tcpO_2_ over the 180 minutes. The secondary endpoints are: time-weighted average of tcpCO_2_, time-weighted average of SpO_2_, time-weighted average of respiratory rate, incidence rate of marked hypercapnia in which tcpCO_2_ is maintained at 60 mm Hg or higher (equivalent to PaCO_2_ >55 mm Hg for 5 minutes or longer), incidence rate of moderate hypercapnia with tcpCO_2_ of 50 mm Hg or more (equivalent to PaCO_2_ >45 mm Hg), percentage of SpO_2_ that fell below 90% and required oxygen administration. The weighted average partial pressure will be calculated, which is the weighted average value for 3 hours (180 minutes), considering the time width between the measurement points.

### 2.8 Safety

The safety evaluation indices of this study are as follows: adverse events are any undesired or unintended signs (including abnormal laboratory values, abnormal vital signs), symptoms or illnesses that occur between the start of medical device use and the end of the last observation. It does not matter whether there is a clear causal relationship or not. Symptoms and diseases occurring before the use of medical devices will be treated as complications and not adverse events. However, if the complications worsen after the date of starting medical device use, they will be treated as adverse events, and the day on which the deterioration is confirmed will be the date of occurrence of the adverse events.

### 2.9 Data collection and management

The assignment table and input table used in this study were created with Research Electronic Data Capture (REDCap). The study will be conducted after allocating the registered patients, and the data of all items in the medical record collected in the study is to be assigned to the researcher with the ID entered by physician, co-doctor and co-worker. The principal investigator or co-researcher will approve the input observation/inspection/evaluation data of each research subject immediately after confirming the content. For the data entered in the case report, the principal investigator and the Clinical Research Center Data Management staff will perform a visual check and a logical check. Consequent to each check, if there are any issues or errors in the data, the principal investigator or the research coordinator is to be contacted. The case is fixed by performing data lock on the case when the issue has been resolved, and any modifications have been completed. If there is an error that needs to be corrected after the case is locked, the data management staffs are responsible for overseeing this process.

In this study, monitoring will be carried out in accordance with the research plan and monitoring procedures to ensure that the research is being conducted properly.

### 2.10 Statistical analysis

Since this is an exploratory study, no hypothesis testing is conducted. Point estimates and their 95% confidence intervals are calculated for the efficacy endpoints. The calculated estimates are used to calculate the sample size of the main study.

## 3 Discussion

Patients undergoing surgery under general anesthesia are often given oxygen through a nasal cannula or face mask to prevent hypoxemia in the general ward during the postoperative acute period. As a result, respiratory management can be performed so that the value of percutaneous arterial oxygen saturation is maintained >90%. However, postoperative acute respiratory management should be to maintain normal respiratory function and gas exchange with normal range of CO_2_, and not to merely maintain apparent SpO_2_. Furthermore, it is necessary to be careful when administering a high concentration of oxygen to patients, and if the oxygen level is raised, there is a risk of developing secondary respiratory complications.

In a recent study, NHF with no supplemental oxygen proved to be effective as a respiratory support method under spontaneous breathing during moderate propofol sedation. It has been reported that the partial pressure of transcutaneous CO_2_ and SpO_2_ can be maintained in the normal range by reducing the breathing effort.^[6]^ Therefore, it is possible that NHF without the combined use of oxygen can be effective for patients who are in the state of somnolence or sedation due to the effects of anesthetics during the postoperative acute phase.

If NHF with room air and no supplemental oxygen can prevent hypoxemia and hypercapnia, the authors believe that safer respiratory management will be possible in general wards. NHF can reduce the respiratory effort^[5,9,10]^ and can improve the ventilation during propofol sedation.^[6]^ Furthermore, it has become clear that NHF facilitates saliva swallowing during respiratory management.^[11]^ Recently, Leone et al reported that the use of either noninvasive positive pressure ventilation or continuous positive airway pressure would be preferred to conventional oxygen therapy such as nasal cannula or face mask (<15 L/min) on the basis of a systematic review of the literature.^[12,13]^ They also noted that the NHF may have an advantage in terms of patient tolerance compared to other noninvasive respiratory support techniques. They strongly advocated for further investigation into the efficacy and safety of noninvasive respiratory support during the perioperative/periprocedural period, because there has been only one RCT comparing continuous positive airway pressure and conventional oxygen on the ward.^[14]^ This study will provide timely and important information on the efficacy and safety of NHF therapy in the general ward after surgery under general anesthesia.

In the study, NHF that administers room air with no supplemental oxygen is proposed for the respiratory management in the postoperative acute phase of patients undergoing oral surgery under general anesthesia. Also, as there are no previous studies related to this purpose, the valid evaluation items that indicate efficacy are unknown. In addition, there is no basis for calculating the sample size. Therefore, this research will be conducted as an exploratory study, and the verification research will be carried out as the next stage based on the results. Included in the current study is a group in which postoperative management is performed only by spontaneous breathing without performing respiratory support such as oxygen administration. This is designed to investigate whether spontaneous breathing alone will not result in hypoxemia and is safe, as well as the efficacy and necessity of conventional oxygen administration.^[3]^ This exploratory study will evaluate the use of NHF without supplemental oxygen as an effective respiratory support during the acute postoperative period.

## Acknowledgment

The authors thank the colleagues and staff at the Department of Dental Anesthesiology at Nagasaki University Hospital for their support.

## Author contributions

SK, GM, MS, SS, MP, ST and TA are responsible for conceiving and designing the trial, planning data analysis, drafting the manuscript and approving the final manuscript. SK will participate in data collection and is in charge of the recruitment and treatment of patients. All authors will have access to the interim results as well as the capacity to discuss, revise and approve the final manuscript.

**Conceptualization:** Takao Ayuse.

**Writing – review & editing:** Takao Ayuse.
